# Sarcopenia and Its Implications for Metabolic Health

**DOI:** 10.1155/2019/8031705

**Published:** 2019-03-06

**Authors:** Gary R. Hunter, Harshvardhan Singh, Stephen J. Carter, David R. Bryan, Gordon Fisher

**Affiliations:** ^1^Department of Nutrition Science, University of Alabama at Birmingham, Birmingham, AL, USA; ^2^Department of Physical Therapy, University of Alabama at Birmingham, Birmingham, AL, USA; ^3^Department of Kinesiology, School of Public Health–Bloomington, Indiana University, Bloomington, IN, USA; ^4^Department of Human Studies, University of Alabama at Birmingham, Birmingham, AL, USA

## Abstract

Sarcopenia not only affects the ability to lead an active lifestyle but also contributes to increased obesity, reduced quality of life, osteoporosis, and metabolic health, in part due to reduced locomotion economy and ease. On the other hand, increased obesity, decreased quality of life, and reduced metabolic health also contribute to sarcopenia. The purpose of this mini-review is to discuss the implications sarcopenia has for the development of obesity and comorbidities that occur with aging.

## 1. Introduction

Aging is related to changes in muscle quantity and quality [1], both of which have important implications for functional performance [2]. These adverse changes in muscle quantity, muscle quality, and the resulting functional deficits are referred to under a common name of “sarcopenia.” Coined first by Rosenberg [3], sarcopenia has undergone changes in it is operational definition. Baumgartner et al. [4] used the relative skeletal muscle mass index which is calculated by normalizing appendicular skeletal muscle mass, measured by dual-energy X-ray absorptiometry, to height squared in meters to diagnose sarcopenia. The European Working Group on Sarcopenia in Older People (EWGSOP) recommends diagnosis of sarcopenia based on the presence of low muscle mass and low muscle strength or low physical performance [5]. Notably, sarcopenia, as an independent medical condition, is now classified under the International Classification of Diseases, Tenth Revision, Clinical Modification (ICD-10-CM), code by the Centers of Disease Control and Prevention [6]. For the purposes of this paper, we define sarcopenia as originally defined by Rosenberg [3], an age-related loss of muscle, and view the functional aspects of the most recent definitions as consequences of sarcopenia. We acknowledge, however, that loss of muscle does not account for all the loss of function experienced by older adults.

**Figure 1:**
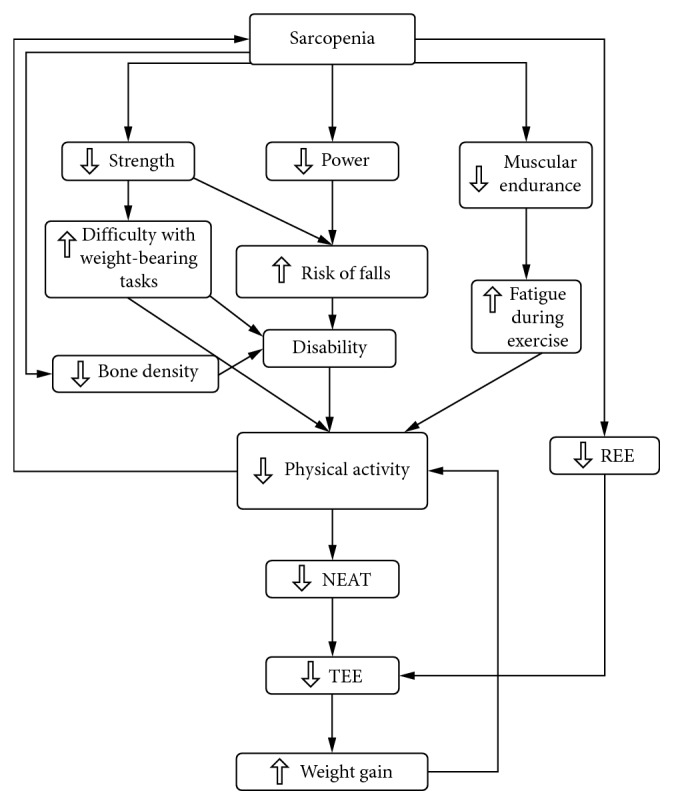
Model describing how sarcopenia affects NEAT and weight gain.

Sarcopenia is associated with decreased mobility [7], lower muscle performance [2], and poorer metabolic health [8]. Moreover, sarcopenia is associated with decreases in ease of locomotion, resting energy expenditure (REE), nonstructured free living physical activity (physical activity not associated with exercise training, termed NEAT), and increased fat mass [9], and all factors that have been linked to obesity and metabolic health. Evidence suggests that loss of muscle fiber size and number accelerates after the fifth decade of life [10]. Only small muscle losses appear prior to 50 years (<10%), whereas 30–40% of muscle is lost between 50 and 80 years [9]. Loss of muscle both directly (because of storing energy previously contained in atrophying muscle as fat, explained in Section 3) and indirectly (because of low total energy expenditure) can increase fat storage. Further compounding the loss in function with age is preferential loss of cross-sectional area of stronger and faster contracting type II muscle fibers [9].  Figure 1 outlines our concept of how sarcopenia increases the risk of obesity and poor metabolic health. A clearer understanding of the relationship between sarcopenia and chronic diseases will help us to design optimal rehabilitation measures to address sarcopenia and associated problems with aging. Thus, the purpose of this paper is to discuss the implications sarcopenia has for the development of obesity and comorbidities that occur with aging.

## 2. Potential Causes of Sarcopenia

Causes of sarcopenia can be grouped under the following primary categories: (a) senescence of satellite cells; (b) oxidative stress; (b) chronic inflammation, and (d) loss of motor units.

### 2.1. Senescence of Satellite Cells

Skeletal muscle contains a number of different progenitor cells, including satellite and mesenchymal progenitor cells. Regeneration of muscle after injury normally occurs rapidly with satellite stem cells that reside in the space between the basal lamina and the sarcolemma, playing a major role [11]. A number of different local transcription factors called myogenic regulatory factors (MRFs), including MyoD, myf-5, myogenin, and myf-6, stimulate proliferation and differentiation of satellite cells [9]. In addition, insulin, such as growth factor (IGF) 1Ea and/or IGF-1Ec (MGF), also stimulates proliferation and differentiation of satellite cells. On the other hand, myostatin has an inhibitory effect on proliferation and differentiation of satellite cells. With injury, the normally inactive satellite cells proliferate into myoblasts and fuse to form myotubes [12, 13]. Senescence of satellite cells, which seems to occur with aging, decreases the ability of the satellite cells to proliferate, thereby reducing their ability to repair skeletal muscle. Mesenchymal progenitor cells normally support satellite cells and promote muscle regeneration. In aging, mesenchymal progenitor cells may become senescent and be involved with the infiltration of fibrous and adipose tissue into skeletal muscle. While the precise mechanisms remain to be elucidated, it is possible that the loss of function of the mesenchymal progenitor cells via oxidative stress may independently and/or via upstream regulation of satellite cells play a role in the decline in regeneration potential and atrophy of skeletal muscle during aging [14].

### 2.2. Oxidative Stress

Larger percentage of type II (especially type IIx) and smaller percentage of type I muscle fiber are related to poorer metabolic health as evidenced by increased blood pressure, increased LDL cholesterol, decreased insulin sensitivity, and decreased arterial elasticity [15]. Type II muscle fibers are more closely associated with higher oxidative stress than type I muscle fibers [16, 17], suggesting the possibility that the observations between myofiber type may be mediated by differences in mitochondria content/function and/or redox homeostasis. Furthermore, immobilization studies have shown reduced mitochondrial content and subsequent increases in mitochondrial reactive oxygen species production following 14 days of quadriceps immobilization [18]. However, studies assessing the role of cellular senescence on age-related loss of musculoskeletal health have shown increases in mitochondria mass and greater ROS production in senescent cells [19]. While the exact mechanisms are not entirely clear, it has been shown that the ATP production via oxidative phosphorylation is reduced; thus although mitochondrial mass is greater, it appears that the actual function of the mitochondria is impaired [19, 20]. Thus, it is possible that the impaired mitochondrial function within type II muscle fibers may induce greater reactive oxygen species production leading to oxidative stress within the muscle. This increased oxidative stress can in turn cause damage to mitochondria leading to an apoptotic cascade that leads to DNA fragmentation, removal of nuclei, and cell death [21]. Alpha motor neuron death also can occur following oxidative stress with the removal of the neuron nuclei [21]. Since older adults have been shown to have decreased type II muscle fiber area compared to younger adults [22], it is unlikely that increases in type II muscle fiber are responsible for the aging-related increase in metabolic health risk. However, it is possible that mitochondrial dysfunction and increased ROS production within type II muscle fibers contribute to the onset of age associated chronic metabolic diseases. Thus, given that resistance training seems to decrease the percentage of type IIx muscle fiber similarly [22] in both young and old, exercise training may be important for older adults in decreasing oxidative stress and maintaining skeletal muscle metabolic function. Oxidative stress can also adversely affect mitochondria including mitochondrial DNA [23]. Interestingly, mitochondrial abnormalities, including mitochondrial DNA deletions, have been reported to increase with aging [24, 25].

### 2.3. Chronic Inflammation

**Figure 2:**
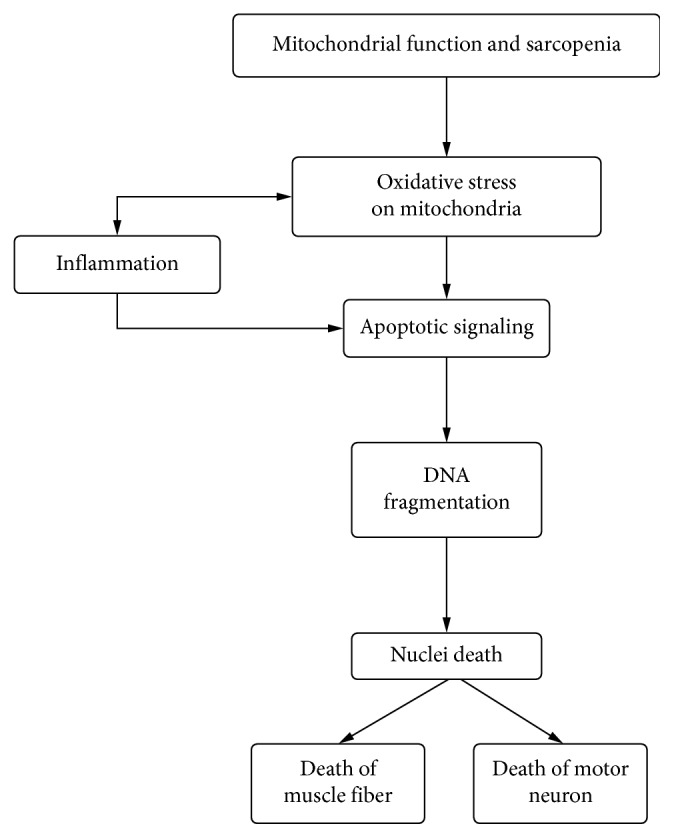
Model for hypothesis that oxidative stress and chronic inflammation influence muscle loss.

Another factor that may influence muscle growth/atrophy is chronic inflammation. We have previously found that older women who have elevated plasma concentrations of TNF-*α* both prior to training and after training did not experience muscle hypertrophy during a 16 week resistance training program. This study did not contain a control group that did no exercise training, so it is possible that differences in individual muscle responses to the training may have been influenced by variability in test results across time [26]. However, those individuals who had “normal” TNF-*α* concentrations were successful in increasing muscle size [27]. While the exact factors responsible for the potential interference in skeletal muscle remodeling remain to be elucidated, low-grade chronic inflammation is likely a contributor [28, 29]. For example, a number of rodent studies have shown the ability of proinflammatory cytokines (CRP, IL-6, and TNF-*α*) to increase proteolysis and increase muscle atrophy [3, 4, 7]. Furthermore, inhibition of TNF-*α* is able to attenuate muscle proteolysis in rodents, whereas infusion of TNF-*α* increased myofibrillar protein breakdown [2, 7]. Additionally, a number of studies have shown independent associations between circulating CRP concentrations and loss of lean body mass and age-related muscle loss [1, 5]. Thus, as depicted in Figure 2, inflammation and oxidative stress appear to be primary factors in the etiology of muscle loss and may also blunt exercise-induced improvements in muscle size and function [30].

### 2.4. Loss of Motor Units

It has been reported that there is a greater loss in number and area of Type II versus Type I motor units with aging [10, 31]. It is unknown at this time whether the loss is due to loss of motor units or reinnervation to type I muscle fibers, although it is probable that it may be some of each. There is a progressive increase in loss of functional motor units with aging [32]. This may occur due to interplay of various factors such as: (a) increased oxidative stress of motoneurons with aging and (b) increased oxidative stress of compensatory innervation of the denervated muscle fibers [33]. Compensatory innervation is associated with the expansion of motor unit size. Notably, enlarged functional motor units become nonoptimal with aging and lead to recession of motor units [33]. Moreover, along with an increase in size of the motor unit, a decreased motor unit firing rate for tasks comparable to daily mobility has been reported in older adults [34]. Thus, a progressive decline in neuromuscular efficacy [35] diminishes the ability of motor units to sustain established connections between nerve and muscle [33]. In addition, aging-associated deterioration of axons leads to an incremental deficit in quantity of functional motor units. Interestingly, a preferential denervation of fast motor units could lead to a greater loss in number and area of Type II versus Type I motor units with aging [36]. However, muscle disuse can adversely affect Type I motor units to a greater degree than Type II motor units [37]. Interestingly, the initial loss of motor units with aging has minimal effects on the function because of compensatory innervation of muscle fibers which lost motor units [38]. This reinnervation typically occurs from a larger and slower motor unit [39]. Clinical observations of slower functional performance such as slower gait speed [40] and reduced muscle power [41] with aging corroborate the findings of greater proportion of slower motor units with aging. The preferential loss of Type II muscle fibers is mainly responsible for loss in power generating capacity of muscles. The functional implications of loss of muscle power may appear earlier versus loss of muscle strength associated with sarcopenia [2]. Moreover, loss in fast motor units can result in adaptive changes at the spinal cord.

## 3. Sarcopenia and Obesity

Further compounding the age-related loss of muscle is an increase in fat mass. Muscle mass is lower in obese mice [42], and obesity can impair regenerative capacity of skeletal muscles [43]. Moreover, obesity can adversely affect the function of satellite cells present in muscle [43]. Fat mass increases with muscle loss in two potential ways. The most direct way is the storage of the energy contained in atrophying muscle as fat. Skeletal muscle is estimated to contain 1.8 kcal/gram of muscle. When muscle atrophies only 1.1 kcal/g of energy is released [44, 45]. However, the energy contained in the atrophying muscle tissue remains in the body unless negative energy balance ensues. Thus, becomes stored as fat. For example, women on average lose approximately 6 kg of lean tissue (presumably mostly muscle) between the age of 25 and 65 years [46], which would result in an increase of slightly less than one kg of fat. Furthermore, it should be noted that despite, a one kg increase in fat mass, this woman would actually lose 5 kg body weight if she were in energy balance (6 kg loss of lean tissue and 1 kg gain in fat tissue). Thus, given that most men and women gain weight across this time period, it is apparent they must be in positive energy balance. The point that needs to be made is that, under conditions where there is atrophy of muscle, body weight will give a very incomplete understanding of what is happening to body composition.

Another factor affecting age-related loss of muscle and weight gain is a decline in insulin sensitivity. Fat cells and immune cells create a condition of low-grade inflammation [47]. This unfavorable adipokine/cytokine profile further decreases insulin sensitivity, which amplifies inflammation and oxidative stress, but also contributes to ectopic fat disposition [47, 48]. Oxidative stress-derived inflammation may be a major mechanism in the pathogenesis and progression of obesity-related metabolic diseases and sarcopenia [49]. Additionally, a rise in inflammatory cytokine levels may facilitate a further increase in oxidative stress, leading to a vicious cycle [50]. The complex and interrelated association between oxidative stress and inflammation in obesity makes it difficult to determine a sequential cause and effect.

Changes in energy expenditure may impact weight gain. Although the impact REE has on weight gain is relatively small in young adults [51], it is possible the large decreases in FFM and thus REE in older adults can contribute to the increase in fat mass with increased age [52]. We are aware of no observations of resting energy expenditure for a large group of individuals across the lifespan. However, the regression for estimation of REE from age for women 23–77 years is 7.0 kcal/day/year [46, 53], resulting in a 280 kcal/day decrease in REE between the ages of 25 and 65 years. It is well established that increased REE is induced by either aerobic and/or resistance training and that both of these strategies are helpful for maintaining body composition [9, 54]. It is important to point out that resistance training and aerobic training probably increase REE through two different mechanisms. Resistance training affects REE primarily through increased muscle mass since skeletal muscle has an REE approximately three times the equivalent for a given amount of fat. However, aerobic training does little to increase muscle mass. In addition, chronic aerobic exercise training does not seem to increase REE when measured at least 72 hours after the last exercise bout [55]. However, REE is increased for at least 19–22 hours following a bout of aerobic exercise [54–56], especially high intensity exercise. Repair of exercise-induced muscle damage and increased sympathetic tone are likely at least partially responsible for the increased REE following a bout of aerobic exercise [55].

Sarcopenia can also induce gains in fat through reductions in physical activity. Sarcopenia is known to impair the ability to perform physical activity [57]. Reduced physical activity is one of the many factors that can lead to obesity [58], which in turn can amplify reduced physical activity [59]. Reduction in physical activity most often occurs slowly and is not recognized by the individual. Weight gain is related to low levels of energy expenditure, especially activity-related energy expenditure [60, 61]. Total energy expenditure decreases approximately 30% between 25 and 90 years [9]. This is particularly apparent with activity-related energy expenditure, the most variable of the 3 energy expenditure pools (activity-related energy expenditure, resting energy expenditure, and thermogenesis of food). Consistent with the concept that physical activity is important for prevention of fat gain, we have shown that physical inactivity is associated with weight gain while physical activity associates with weight maintenance [9]. For example, women who maintain weight over one year have 43% higher NEAT as measured with doubly labeled water than women who gained weight [60]. The loss of muscle leads to a loss of function and thus ease of physical activity [62]. This decreased ease of movement results in reduced NEAT [51], while reduced NEAT leads to fat gain [60, 61]. Even though sarcopenia only directly leads to about a 1 kg increase in fat mass between 25 and 65 years, it probably contributes to an additional 9 kg gain in fat mass through a loss of function and decreased ease of locomotion that leads to reduced NEAT.

Several factors influence ease and economy of locomotion. Ease of locomotion is markedly affected by aerobic fitness. A more aerobically fit individual will have a lower heart rate and perceived effort while walking, cycling, or running at a certain speed. Muscular strength has also been shown to be associated with locomotion economy and ease of movement [9, 63–65]. A stronger individual walks or runs more economically so is able to do the task with more ease [63, 66, 67]. In addition, retention of muscle during weight loss results in a maintenance of ease and economy in walking [68]. An increase in muscle strength may change the muscle fiber activation pattern, resulting in less dependency on inefficient type II muscle fibers. This would increase locomotion economy and thus ease of locomotion. On the contrary, weight gain can decrease ease of locomotion economy. For example, a sarcopenic obese individual with less muscle has an increased difficulty moving but is also plagued by excess body weight making movement even more difficult.

Another factor that may impact changes in locomotion ease and economy could be the use of stretch shortening cycle potentiation (SSCP) [69, 70]. An individual can jump higher if he/she drops from a small bench prior to the jump as opposed to statically holding the jump position prior to the jump. A good portion of the increased potentiation in the jump probably results from stretch of elastic tissue of muscle and tendon and then use of energy stored in elastic tissue in the muscle/tendon complex during the take-off phase of the jump [70]. The use of the elastic energy increases force and velocity during the following jump, thus increasing jump height. This utilization of elastic energy obtained during a rapid stretch of muscle is termed SSCP. It saves energy since gravity is used to stretch the muscle so the stored elastic energy used in the jump requires little energy expenditure [69]. This has an effect on increasing endurance capabilities (because of the saving of energy expended). The increased force, velocity, and endurance obtained from SSCP are important in almost all sports. In addition, it has also been established that it is important in maintaining ease in walking in nonathletes [71–73]. This is important since ease of walking has been established as one factor that influences free living physical activity [64, 66, 74] and free living physical activity has profound effects on metabolic health. Muscle elasticity can also play a critical role in locomotion. Aging-associated loss in mechanical properties of muscle can adversely affect our ability to utilize the SSCP [75] and thus could lead to decreasing economy and ease of locomotion and participation in free living physical activity, specifically those activities which require generation of muscle power. In other words, older individuals lose the “spring in their step,” which may predispose them to be less active and contribute to their susceptibility for metabolic diseases such as diabetes, heart disease, and cancer.

Thus, it can be argued that SSCP is important for performance of both athlete and nonathlete populations and can indirectly affect metabolic health of everyone. We have previously shown that strength of a muscle is important for utilization of SSCP. Stronger muscles are able to develop force rapidly, stopping the eccentric muscle action (lengthening contraction) and allowing more stretch of the muscle/tendon complex, thus developing more force and velocity during SSCP [76]. Therefore one strategy for improving/maintaining SSCP is increasing muscle strength. We have also shown that those individuals who have a higher percent fast twitch muscle fibers are able to delay eccentric force production until late in the stance phase, develop more eccentric force, and obtain more SSCP [76]. As pointed out earlier, older adults seem to preferentially lose fast twitch muscle fiber volume. Therefore, it may be important to develop strategies for maintaining fast twitch muscle fiber volume so that function can be better maintained as we age.

### 3.1. Sarcopenia and Skeletal Health

Per the mechanostat model, maximum voluntary forces on bone are produced by muscles [77]. Therefore, individuals who place more voluntary forces on their bone such as gymnasts have enhanced bone density [78]. It is postulated that individuals with sarcopenia who have low muscle mass and muscle strength will have weaker bones. This is corroborated by our previous finding of leg press strength as a significant predictor of bone density and bone strength in older adults [79]. Moreover, a positive association has been reported between sarcopenia and osteopenia/osteoporosis, which is a clinical diagnosis of low bone mass [80, 81]. Notably, there is some evidence that adults over the age of 50 and diagnosed concurrently with sarcopenia and obesity have a greater risk for osteoporosis than individuals diagnosed with sarcopenia [82] or obesity [83] only. Importantly, this relationship remains after adjusting for age, sex, and exercise level [82]. There is evidence that increased body mass due to addition of fat tissue to high-muscle phenotype does not confer any additional benefit on BMD [84]. This is not surprising because it is well known that bone density and strength are determined to a great degree by strain magnitude [85] and that, due to loss in muscle mass and muscle strength, strain magnitude is greatly reduced in sarcopenia. Furthermore, the relationship between sarcopenia and osteopenia/osteoporosis may also be driven by age, sex, and race. A previous study which had more than half of its study population as female did not find any relationship between sarcopenia and osteopenia/osteoporosis [79]. This may be due to a greater degree of aging-associated loss of muscle mass in men versus women [86].

### 3.2. Sarcopenia and Metabolic Health

In all probability, low exercise capacity is one of the highest risk factors, if not the highest for all-cause morbidity and mortality [87, 88]. The ability to be physically active (high NEAT) is important for maintenance of metabolic health and weight. Exercise capacity makes it easier to be physically active while physical activity contributes to maintenance of exercise capacity, so it is difficult to separate the two parameters. However, it is likely that exercise capacity (aerobic fitness), physical activity, and low body fat have independent impacts on risk of disease [89, 90]. In addition, all three factors are intimately interrelated and changes in one variable can affect the other two variables. For example, improved fitness may increase physical activity and reduce body fat, while loss of fat may increase ease of locomotion and thus physical activity. It is important to note that although fitness and physical activity are intimately linked, they are different entities. For example, it would be possible to have relatively high levels of NEAT with large amounts of moderate to low intensity physical activity. This would do little to change the amount of muscle or fitness (aerobic or strength). In addition, it would be possible to do a small amount of very intense aerobic training that would increase aerobic fitness but be sedentary the rest of the time, maintaining moderately high fitness but accumulating small amounts of physical activity. Although, it is not clear whether aerobic fitness, physical activity, and/or body composition have the largest impact on metabolic health; it is clear that changes in one of these variables impact the other variables and may impact metabolic health indirectly through that variable. In other words, decreases in physical fitness and activity may affect body composition, and changes in body composition and fitness may influence participation in physical activity, while changes in body composition and physical activity may influence fitness.

As indicated above, another factor that may influence NEAT is the ease of locomotion. For example, several studies have shown that ease of walking is associated with increased NEAT [55, 66, 91]. Aerobic exercise training can increase exercise tolerance through increases in maximal oxygen uptake (VO_2_max) and adaptations in skeletal muscle such as an enhanced mitochondrial oxidative phosphorylation capacity [92]. Although central factors such as the ability of the cardiovascular system to deliver oxygen to the working muscle probably control aerobic capacity (maximum oxygen uptake), mitochondrial function slows the development of fatigue with continued muscle contractions [92]. Enhanced insulin sensitivity and reduced risk of cardiovascular disease result from aerobic exercise training [93]. However, aerobic training normally does not result in large increases in skeletal muscle mass [6] and may make aerobic training a poor strategy for maintenance of muscle mass during aging [68]. We are aware of no controlled studies designed to compare the long-term effects of aerobic and resistance training on age-related muscle atrophy in humans. However, it seems probable to us that those individuals who resist training across the lifespan will have a larger reserve of muscle to lose before they become sarcopenic.

## 4. Exercise Training

Expenditure of energy is 2 to 3 times larger with aerobic training compared to resistance training of similar durations. Therefore, it is beneficial in slowing weight gain. Aerobic training may be better than resistance training for maintaining metabolic health, i.e., reduce oxidative stress and inflammation, increase insulin sensitivity, decrease blood pressure, and improve blood lipid profile [94, 95]. In addition, decreases in oxidative stress and inflammation may slow the rate of muscle loss. Thus, it is advisable for all adults, especially older adults, to participate in at least some form of aerobic training. It should be pointed out that the combination of aerobic and resistance training may be the optimal strategy for increasing insulin sensitivity and function in older adults [96].

**Figure 3:**
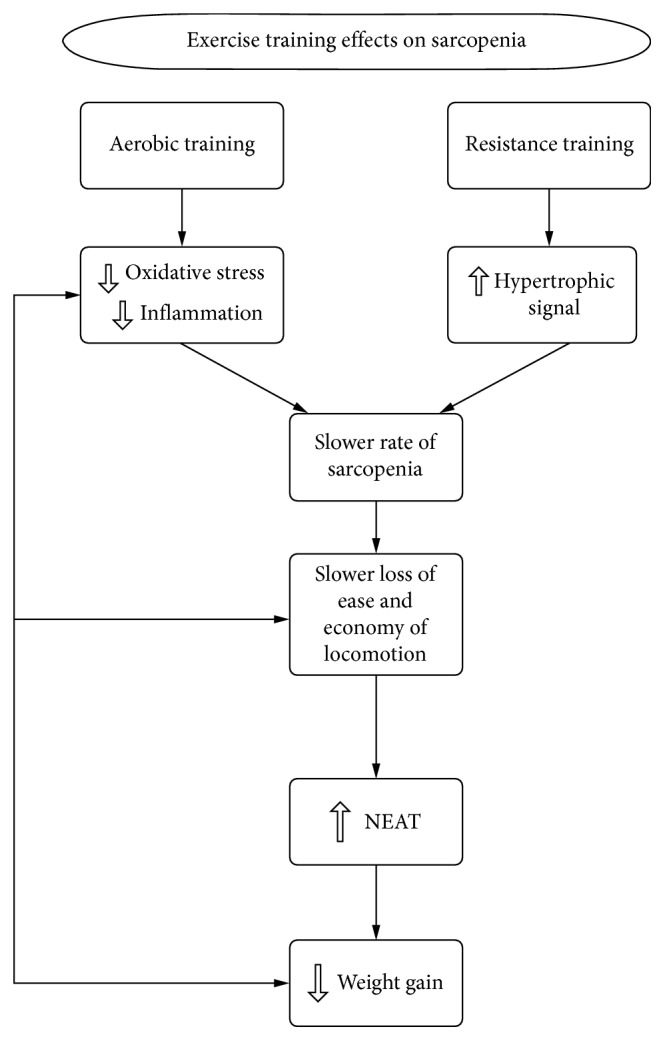
Exercise training effects on sarcopenia.

Although aerobic training is an important factor for improving cardiometabolic health, resistance training is probably the best choice for delaying sarcopenia. Resistance training is well established as an intervention that can increase skeletal muscle mass [9] and in all likelihood slow but not stop the loss of muscle as we age [22]. Decreased skeletal muscle can impair locomotion economy and ease while contributing to reduced NEAT [9, 60], which in turn may increase loss of muscle. Resistance training normally results in increases in muscle mass but little or no change in body weight. A gain in muscle mass with no change in body weight results in a loss of fat tissue. For example, 24 weeks of resistance training in older men and women produced over a 2 kg increase in FFM and a 2.7 kg loss of fat mass resulting in a nonsignificant decrease in body weight but a highly significant 3.4% decrease in percent fat [64]. Thus, despite little or no change in body weight resistance training can lead to increases in lean mass and losses in fat mass. Not only will both aerobic and resistance training make individuals leaner it appears to direct fat away from the viscera [97], a fat depot that is considered to be more damaging to metabolic health than fat in other parts of the body such as the legs. For example, visceral fat is consistently related to increase in cardiovascular risk factors such as cholesterol, blood pressure, and insulin sensitivity, while leg fat is related to reduced risk [98–101], although visceral fat in parous women do not seem to demonstrate as strong a relationship between visceral fat and insulin sensitivity as nonparous women [101]. In addition, resistance training is associated with preferential reduction of visceral fat compared to peripheral fat stores [102]. Aerobic and resistance training also slows the regain in body weight following weight loss and prevents regain of visceral fat for one year following over a 12 kg diet-induced weight loss [97]. The ability of resistance training to prevent visceral fat gain may be particularly the case in older populations as fat seems to shift from the periphery to the viscera as we age [46]. Although diet restriction is necessary for large weight loss and any exercise training program is only practical for small losses of body fat, i.e., 2–4 pounds, exercise training, particularly resistance training, is very beneficial for preventing loss of muscle and fat gain, especially visceral fat.  Figure 3 illustrates our understanding of how exercise training affects sarcopenia and obesity risk in aging populations.

### 4.1. Training Suggestions

Age, gender, genetic predisposition, prior training status, and general health are a few of the factors that influence the optimal training program for increases in muscle mass and strength. So it is not too surprising that there is little consensus for an optimal program for those individuals who are either sarcopenic or vulnerable to sarcopenia. Some general guidelines for older adults have been established based on a meta-analysis by Rhea et al. [103]. It is recommended that the load lifted should be between 60 and 80% of the maximum weight that can be lifted one time (1RM) for 2–4 sets of 8–15 repetitions. Two times/week training may be best for increasing muscle size, strength, ease of locomotion, and NEAT in older adults [65]. Since loss of type II muscle fiber with aging leads to loss of velocity in shortening and power (all factors that may influence ease of locomotion and risk of falls), it may be important to include some high-velocity contractions in training programs. Although it is unknown whether high-velocity training can slow the age-related loss of type II muscle fiber loss, it would seem prudent to include some high-speed training at least once/week.

The position taken in this paper is prevention of sarcopenia can have important implications for participation in NEAT and prevention of obesity. However, it should be pointed out that maintenance of muscle mass probably cannot totally prevent age-related changes in function. Although resistance training is probably more successful at preventing sarcopenia than aerobic training, it is important to note that two to three times as much energy is burned during aerobic exercise compared to an equivalent time spent in resistance exercise. Thus, aerobic exercise also appears to have an independent positive effect on longevity and a number of health-related risk factors [94, 95]. It is our contention that the combination of aerobic and resistance training will slow sarcopenia development, decrease fat mass accretion (especially harmful visceral fat), and decrease the risk of developing a number of metabolic diseases throughout the lifespan.

## Abbreviation

REE: Resting energy expenditure
TEE: Total daily energy expenditure
NEAT: Nonstructured free living physical activity (physical activity not associated with exercise training)
CRP: Proinflammatory cycokine, C reactive protein
IL-6: Proinflammatory cytokine, interleukin 6
TNF-*α*: Proinflammatory cytokine, tumor necrosis factor alpha
SSCP: Stretch shortening cycle potentiation.
